# Partial weight bearing in hip fracture rehabilitation

**DOI:** 10.4155/fsoa-2017-0068

**Published:** 2017-10-12

**Authors:** Sahar Ahmed Abdalbary

**Affiliations:** 1 Lecturer at the Department of Orthopedic Physical Therapy, Faculty of Physical Therapy, Egyptian–Chinese University, Cairo, Egypt

**Keywords:** biofeedback systems, hip fractures, partial weight bearing

Orthopedic rehabilitation is very important for ensuring the best outcomes of treatment for musculoskeletal disorders and disabilities, especially in terms of hip fracture in old-aged patients.

This field of research attempts to maximize the use of biomechanics and biology to improve functional outcomes and the overall well-being of patients.

Orthopedic rehabilitation is a very important part of every musculoskeletal care delivery system. This article highlights recent research and advances in hip fracture in geriatric patients’ rehabilitation.

Hip fractures in older patients are one of the most common injuries; in the USA alone, in 2003, hip fracture cases represented around 30% of all hospitalized cases [1]. Early postoperative improvement has been shown to improve following early ambulation after hip surgery, and early mobility enhances early recovery. When therapists use partial weight bearing, it helps early recovery [2].

Some degree of weight bearing is very important to activate osteoblasts and other cells responsible for bone healing. Further complications may result from immobilization postsurgery. In an attempt to generate a maximum mechanical environment at different stages of fracture healing, many studies prescribe partial weight bearing for lower extremities after a period of nonweight bearing. Partial weight bearing includes increasing weight loading on the limb progressively over time, which varies between patients based on the extent of the injury and the judgment of the clinician [3].

Control of postoperative pain is a vital part of rehabilitation for ensuring patient safety [4].

Partial weight bearing is prescribed for patients following pelvic fracture to protect the healing of bone and/or surgical constructs and provide a stimulus for bone growth. To date, the ability of patients to produce partial weight bearing is attached to their ability to reproduce partial weight-bearing orders; in other words, when the therapist provides clear and comprehensive orders of how much weight should put on the patient's affected limb, this leads to accurate weight bearing and good healing [5].

A study by Yu *et al*. [6] attempted to measure the ability of patients to reproduce partial weight-bearing orders with the hand under foot, bathroom scale and verbal methods of instruction, as well as to determine the effect of partial weight bearing on long-term clinical outcomes. This study concluded that partial weight bearing could not accurately be reproduced with any of the weight-bearing techniques prescribed, which was supported by previous evidence showing an inability to accurately reproduce partial weight-bearing orders.

Biomechanically, nonweight-bearing causes the effective center of gravity to move distally and away from the nonsupporting leg. This increases abductor muscle forces, which results in joint compressive forces that are several times the body weight [7].

Consequently, partial weight bearing or at least toe-touch weight bearing is favorable. The use of biofeedback devices seems promising to support weight-bearing instructions. Smart steps and biofeedback devices could provide real-time feedback by enabling the therapist to determine accurately what is the weight bearing that can be applied for the patient and when they can increase the weight-bearing load [8].

Accelerated rehabilitation following hip fracture comprises early unrestricted weight bearing and muscle strengthening. The effect of crutches, an orthotic garment and strapping system, TheraTogs and no walking aids over 3–4 weeks on walking speed, trunk sway and muscle activity have been examined. All measured parameters increased in the TheraTogs phase more than in the crutches or no-aids phases. This may be because muscle activity was facilitated, enabling active support of recovering structures [9].

In an observational study, patients with hip fracture were monitored 1 day per week with the Feet B@ck system during their admission and after 1 week. Outcome measures of the Feet B@ck system are steps, walking bouts and loading rate. The study concluded that the loading rate is a sensitive weight-loading parameter for analysis of dynamic weight loading during rehabilitation in old patients with hip fractures and this parameter correlates with clinical improvement [10].

Alternatively, aquatic therapy is an ideal method for early mobilization, allowing graduated increase in lower extremity weight bearing due to a decrease in displacement with depth of immersion. Since the water provides a partial weight-bearing environment, upper extremity muscle mass is not required to protect the lower extremity [9].

To make predictions for the most effective method for partial weight bearing, we must discuss partial weight-bearing training in terms of two questions that deserve attention:

- What limitations in weight provide the best clinical improvement?
- What is the best method to train patients to comply with weight-bearing instructions?

Weight-bearing loads are partially affected by the surgeon's choice of a conservative approach to weight bearing with respect to patient tolerance.

Adequate training needs to be initiated before we can expect that patients will comply with weight-bearing instructions, considering pain and fatigue. Thus, in order to better determine the proper ambulation of patients following pelvic fracture, researchers need to better define weight-bearing classifications and find improved methods to rehabilitate patients.

Physical therapists commonly train patients to comply with weight-bearing instructions, utilizing clinical techniques as well as devices that can include scales, biofeedback systems and force plates.

Future research should focus on defining the suitable quantity of partial weight bearing and the best way to use biofeedback devices and include the investigation of outcomes of weight-bearing strategies. With the best-designed studies, this area of research has the potential to improve care for the large majority of lower extremity, weight-bearing orthopedic patients.
